# Diagnostic performance of zero-TE lung MR imaging in FDG PET/MRI for pulmonary malignancies

**DOI:** 10.1007/s00330-020-06848-z

**Published:** 2020-04-16

**Authors:** Feibi Zeng, Munenobu Nogami, Yoshiko R. Ueno, Tomonori Kanda, Keitaro Sofue, Kazuhiro Kubo, Takako Kurimoto, Takamichi Murakami

**Affiliations:** 1 Department of Radiology, Kobe University Graduate School of Medicine, 7-5-2 Kusunoki-cho, Chuo-ku, Kobe, Hyogo 650-0017 Japan; 2 GE Healthcare, Hino, Tokyo Japan

**Keywords:** Lung, Fluorodeoxyglucose F18, Magnetic resonance imaging, Positron-emission tomography

## Abstract

**Objectives:**

This study aimed to evaluate the diagnostic performance of the lung zero-echo time (ZTE) sequence in FDG PET/MRI for detection and differentiation of lung lesions in oncologic patients in comparison with conventional two-point Dixon-based MR imaging.

**Methods:**

In this single-institution retrospective study approved by the institutional review board, 209 patients with malignancies (97 men and 112 women; age range, 17–89 years; mean age, 66.5 ± 12.9 years) underwent ^18^F-FDG PET/MRI between August 2017 and August 2018, with diagnostic Dixon and ZTE under respiratory gating acquired simultaneously with PET. Image analysis was performed for PET/Dixon and PET/ZTE fused images by two readers to assess the detectability and differentiation of lung lesions. The reference standard was pathological findings and/or the data from a chest CT. The detection and differentiation abilities were evaluated for all lesions and subgroups divided by lesion size and maximum standardized uptake value (SUVmax).

**Results:**

Based on the reference standard, 227 lung lesions were identified in 113 patients. The detectability of PET/ZTE was significantly better than that of PET/Dixon for overall lesions, lesions with a SUVmax less than 3.0 and lesions smaller than 4 mm (*p* < 0.01). The diagnostic performance of PET/ZTE was significantly better than that of PET/Dixon for overall lesions and lesions smaller than 4 mm (*p* < 0.01).

**Conclusions:**

ZTE can improve diagnostic performance in the detection and differentiation of both FDG-avid and non-FDG-avid lung lesions smaller than 4 mm in size, yielding a promising tool to enhance the utility of FDG PET/MRI in oncology patients with lung lesions.

**Key Points:**

• *The detection rate of PET/ZTE for lesions with a SUVmax of less than 1.0 was significantly better than that of PET/Dixon.*

• *The performance for differentiation of PET/ZTE for lesions that were even smaller than 4 mm in size were significantly better than that of PET/Dixon.*

• *Inter-rater agreement of PET/ZTE for the differentiation of lesions less than 4 mm in size was substantial and better than that of PET/Dixon.*

**Electronic supplementary material:**

The online version of this article (10.1007/s00330-020-06848-z) contains supplementary material, which is available to authorized users.

## Introduction

^18^F-Fluorodeoxyglucose (FDG) PET/CT currently plays a major role in ensuring accurate clinical classification of disease stages and significantly influences therapeutic decisions in the oncologic setting [1, 2]. In this context, PET/MRI is an emerging modality that enables simultaneous acquisition of metabolic information with PET and morphological information with high soft-tissue contrast using MRI. PET/MRI also offers diagnostic advantages over PET/CT for certain kinds of malignancies, including prostate cancer and bone metastasis [3]. Although PET/MRI is reportedly comparable to PET/CT for lung cancer staging [4], a major drawback of PET/MRI using conventional two-point Dixon-based MR imaging is the difficulty in lung MR imaging in comparison with CT imaging [5, 6], which is mainly attributable to the low proton density in the lung, in addition to respiratory and cardiac motions in the thorax [7].

The zero echo time (ZTE) MR sequence is a sequence with minimum susceptibility effect; hence, it is used as a problem-solving method for lung imaging on MR that can provide high-resolution structural information for the lung with a better signal-to-noise ratio and contrast-to-noise ratio due to its very short echo time [8]. ZTE may, therefore, overcome the drawbacks of PET/MRI for the diagnosis of lung lesions.

Our hypothesis was that PET/MRI in combination with lung ZTE was superior to conventional PET/MRI using two-point Dixon in diagnosing lung lesions. The purpose of our study was to evaluate the diagnostic performance of lung ZTE on FDG PET/MRI for the detection and differentiation of lung lesions by comparing these findings with those obtained using conventional two-point Dixon-based MR imaging.

## Materials and methods

### Study inclusion criteria

**Fig. 1:**
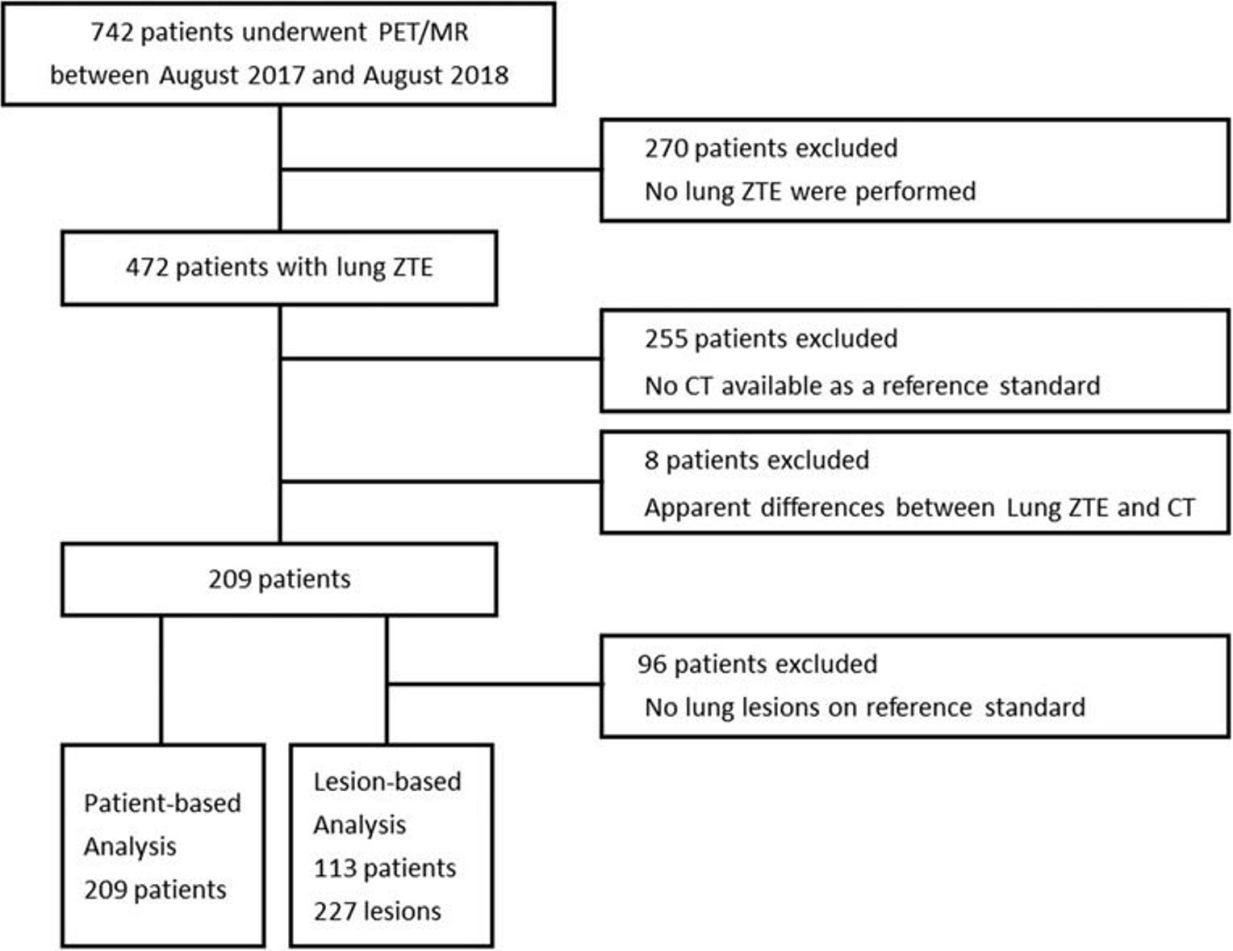
Flowchart of the inclusion criteria of the study

The institutional review board approved this retrospective study and waived the requirement for informed patient consent. Seven hundred and forty-two consecutive patients with proven malignancies underwent ^18^F-FDG PET/MR imaging between August 2017 and August 2018. Excluded were patients who did not undergo lung ZTE (*n* = 270), those with no CT data available as a reference standard (*n* = 255) and those showing apparent differences in lung findings between PET/MRI and CT examinations due to acute inflammatory change (*n* = 8). After application of the exclusion criteria, 209 patients (97 men and 112 women; age range, 17–89 years; mean age, 66.5 ± 12.9 years) were finally enrolled in the patient-based study. Among these 209 patients, 113 (52 men and 61 women; age range, 25–89 years; mean age, 67.8 ± 12.8 years) were enrolled in the lesion-based study after excluding 96 patients who showed no lung lesions on the reference standard (Fig. 1).

### PET/MR imaging protocol

All patients fasted for at least 6 h before the examinations, and blood glucose levels were confirmed to be below 180 mg/dL at the time of injection. Images were acquired on a hybrid PET/MRI scanner (SIGNA PET/MR, GE Healthcare) at 3.0 T in the field strength of the MR component with a 16-channel anterior coil and a 16-channel central molecular imaging array coil. All whole-body PET/MRI examinations were performed 60 min after intravenous injection of 3.5 MBq/kg of ^18^F-FDG. PET images were reconstructed using time-of-flight ordered subset expectation maximization (TOF-OSEM) with two iterations, 16 subsets and a Gaussian filter of 4.0 mm with a point-spread function. A PET emission scan for the thoracic bed was performed for 5 min and respiratory gated by the quiescent period gating (Q. Static) with offset/acquisition windows of 30/50%, respectively. Attenuation correction was performed by a simultaneously acquired two-point Dixon three-dimensional volumetric interpolated fast spoiled gradient echo (Dixon) sequence under free-breathing.

The diagnostic MR imaging technique for the thoracic bed consisted of the Dixon and ZTE sequences with respiratory gating, which were simultaneously acquired with the PET emission scan. No contrast-enhancing material was administered intravenously for MR imaging. Dixon is a three-dimensional dual-echo GRE sequence used with a two-point Dixon method for water-fat separation [9] with the following parameters: repetition time (TR), 4.6 ms; first echo time (TE), 1.3 ms; second TE, 2.6 ms; slice thickness, 4.0 mm; flip angle (FA), 12°; number of excitations (NEX), 1; matrix size, 300 × 200; field of view (FOV), 45.0 cm with 80% phase field of view; approximate acquisition time, 1 min 15 s. ZTE was acquired by 3D radial sampling to provide an isotropic, large field of view and minimal TE of zero [10] with the following parameters: TR, 740–1480 ms; TE, less than 0.02 ms; slice thickness, 2.5 mm; FA, 1°; NEX, 1; matrix size, 320 × 320; FOV, 32.0 cm with 100% phase field of view; number of spokes per segment, 384; approximate acquisition time, 5 min.

Both Dixon and ZTE scans were performed using a respiratory gating method based on MR navigator echoes with an acquisition/acceptance window of 40%/2.0 mm, respectively. In order to avoid any predisposition in PET, the respiratory motion needs to be monitored and compensated in MRI while the scanned subjects are allowed to breathe freely during the simultaneous acquisition of PET and MRI. Breath-hold MRI, despite its resiliency to the motion, is less than ideal for PET/MRI regardless of the MR acquisition method and was therefore omitted from our consideration.

### Image analysis

Image analysis was performed to assess the detectability and differentiation of lung lesions by evaluating PET/Dixon and PET/ZTE fused images on the workstation (Advantage Workstation 4.7, GE Healthcare). Reader 1 (5 years of experience, training in both radiology and nuclear medicine) and reader 2 (21 years of experience, board-certified in both radiology and nuclear medicine) independently interpreted these images without access to patient history. The differentiation of benign and malignant lung lesions was evaluated using a five-point visual score (differentiation score) as follows: (1) definitely benign, (2) probably benign, (3) equivocal, (4) probably malignant and (5) definitely malignant. The criteria for differentiation included morphological findings on MRI, including round, oval, spiculated, cavitated and ground-glass opacity (GGO) based on the CT criteria of the lung nodules [11] as well as the degree of FDG uptake on PET.

In the patient-based analysis, patients with lung lesions showing differentiation scores of 1 to 3 or no detectable lung lesions were considered to have benign disease. In contrast, patients showing lung lesions with differentiation scores of 4 or 5 were considered to have malignant disease. In the lesion-based analysis, assessment of the detectability of lung lesions was performed using a five-point visual score (detectability score) as follows: (1) not detectable, (2) almost undetectable, (3) equivocal, (4) almost detectable, (5) detectable. The differentiation of benign and malignant lung lesions was also evaluated by using the differentiation score.

### Reference standard

The reference standard for image analysis was acquired through separately performed dedicated chest CT examinations within 1 month of the PET/MR examination, and the sizes of the lung lesions were determined. If more than five lesions per patient were found in the reference standard, five lesions were randomly selected by a random number table to avoid clustering bias in the subsequent statistical analysis. Differentiation between benign and malignant cases was performed on the basis of pathological findings acquired during an operation (*n* = 3) and/or bronchoscopy (*n* = 1) and/or follow-up examinations (*n* = 205) at least 16 months (mean 21.8 months, range 16–28 months) after PET/MRI, including CT, PET/CT and PET/MRI. After blinded assessment of the detectability and differentiation of lung lesions independently, the readers matched the lesions by ascertaining images. The lesions without PET/MRI findings were given differentiation and detectability scores of 1.

Reader 1 measured the longest diameter of the lung lesions on CT and classified them into 4 categories (mass, nodule, pure ground-glass opacity and subsolid nodule) depending on the radiological characteristics according to the definitions outlined by the Fleischner Society [11]. The lung lesions were also classified into four subgroups according to the size as follows: smaller than 4 mm, at least 4 mm but smaller than 6 mm, at least 6 mm but smaller than 8 mm and at least 8 mm. Further, reader 1 measured the maximum standardized uptake value (SUVmax) of all the lung lesions on PET images that corresponded to each reference-standard lesion using a manually adjusted cubic volume of interest and classified them into 3 subgroups according to SUVmax (less than 1, non-avid; between 1 and 3, intermediate; larger than 3, avid lesion). SUVmax of the lung lesions found on the reference-standard CT, but not on PET/MRI (both Dixon and ZTE), were determined to be zero.

### Statistical analysis

All statistical analyses were performed by using MedCalc version 18.11 software (MedCalc Software Ltd.). To avoid a clustering bias due to multiple lesions per patient, *p* values of < 0.05 and < 0.01 defined statistically significant differences for the patient-based and lesion-based studies, respectively. The Wilcoxon’s signed-rank test was used to evaluate differences in the detectability of lung lesions between PET/Dixon and PET/ZTE in assessments by each reader for all lesions and subgroups. Pairwise comparison of receiver operating characteristic (ROC) curve analysis was performed to compare differentiation performance by the area under the curve (AUC). Inter-rater variability for PET/Dixon and PET/ZTE was assessed by weighted Cohen’s kappa coefficients. The kappa value was interpreted according to Landis and Koch criteria as follows: poor agreement, less than 0; slight agreement, between 0.00 and 0.20; fair agreement between 0.21 and 0.40; moderate agreement, between 0.41 and 0.60; substantial agreement, 0.61 and 0.80; almost perfect agreement, between 0.81 and 1.00 [12]. All values are shown as mean ± standard deviations unless otherwise specified. To reduce potential bias, one of the co-authors was responsible for the ZTE sequence preparation for the PET/MR scanner and did not participate in any other study-related activity, including lesion assessments and statistical analyses.

## Results

The primary malignancies of these 209 patients were as follows: genitourinary cancer, 59; hepato-biliary-pancreatic cancer, 50; gastrointestinal cancer, 48; head and neck cancer, 30; carcinoma of unknown primary origin, 7; sarcoma, 4; lymphoma, 3; melanoma, 2; squamous cell carcinoma of the skin, 2; lung cancer, 2; gastrointestinal stromal tumour, 1; neuroendocrine tumour, 1. There were a total of 227 lung lesions in 113 patients (median and mean number of lesions per patient, 2 and 2.14, respectively; range, 1–5). The breakdown of the CT findings for these lung lesions was as follows; mass, 2; nodule, 196; pure ground-glass opacity, 27; and subsolid nodule, 2. The mean CT size of all lung lesions was 6.15 ± 5.03 mm (range 1.1–30.3 mm). The mean PET SUVmax of all lung lesions was 1.96 ± 3.51 (range 0–20.57). On the basis of the reference standard, 173 and 54 of 227 lesions were benign and malignant, respectively. The differentiation of four lesions was pathologically confirmed, while 223 lesions were determined by at least 16 months of follow-up. The malignant lesions included 52 metastatic lung tumours and two lung cancers. The number and size of the lesions according to the reference standard and the SUVmax of the lung lesions in the overall lesions and in each subgroup are summarized in Table 1.

**Table 1:** Characteristics of the lung lesions

Group	Number (benign/malignant)	Mean CT size ± SD (mm)	Mean PET SUVmax ± SD
All lung lesions	227 (173/54)	6.15 ± 5.03 (1.1–30.3)	1.96 ± 3.51 (0–20.57)
SUVmax < 1	117 (105/12)	4.81 ± 3.93 (1.5–30.3)	0.50 ± 0.34 (0–0.99)
SUVmax 1–3	78 (57/21)	6.57 ± 4.98 (1.1–26.6)	1.47 ± 0.48 (1.00–2.83)
SUVmax ≥ 3	32 (11/21)	10.1 ± 6.49 (3.6–30.2)	8.44 ± 6.17 (3.19–20.57)
Size < 4 mm	89 (76/13)	2.95 ± 0.65 (1.1–3.9)	0.70 ± 0.58 (0–3.95)
Size 4–6 mm	65 (47/18)	4.82 ± 0.59 (4.0–5.9)	1.35 ± 1.57 (0–10.38)
Size 6–8 mm	32 (19/13)	6.70 ± 0.54 (6.0–7.9)	2.45 ± 2.02 (0–6.97)
Size ≥ 8 mm	41 (31/10)	14.78 ± 6.24 (8.2–30.3)	5.25 ± 6.81 (0–20.57)

Numbers in parentheses for the mean CT size and mean PET SUVmax are range of standard deviation (SD) values

### Patient-based analysis for differentiation

**Fig. 2:**
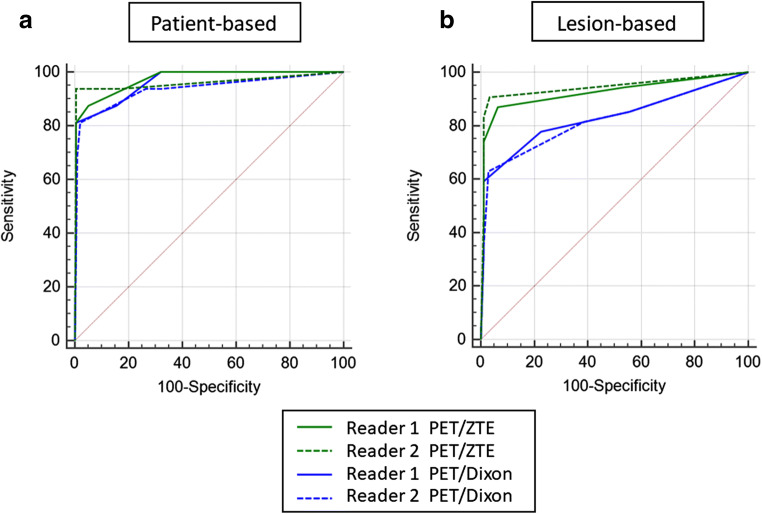
Graphs showing pairwise comparison of receiver operating characteristic (ROC) curve analysis to assess the diagnostic performance in differentiating the overall lung lesions by each method, which is represented as the areas under the curve (AUCs) for patient-based (**a**) and lesion-based (**b**) analyses. PET/ZTE showed larger AUC than PET/Dixon for both readers and provided statistically significant difference for reader 2 for patient-based analysis (*p* < 0.05). In lesion-based analysis, PET/ZTE showed significantly higher diagnostic performance than PET/Dixon for both readers 1 and 2 (*p* < 0.01)

The ROC curves are shown in Fig. 2. The AUC of PET/ZTE was significantly larger than that of PET/Dixon in assessments by reader 2 (PET/Dixon, 0.935; PET/ZTE, 0.961; *p* = 0.0451). In contrast, there was no statistical difference in the assessments by reader 1 (PET/Dixon, 0.962; PET/ZTE, 0.972; *p* = 0.1080).

### Lesion-based analysis: detectability of the lung lesions

**Fig. 3:**
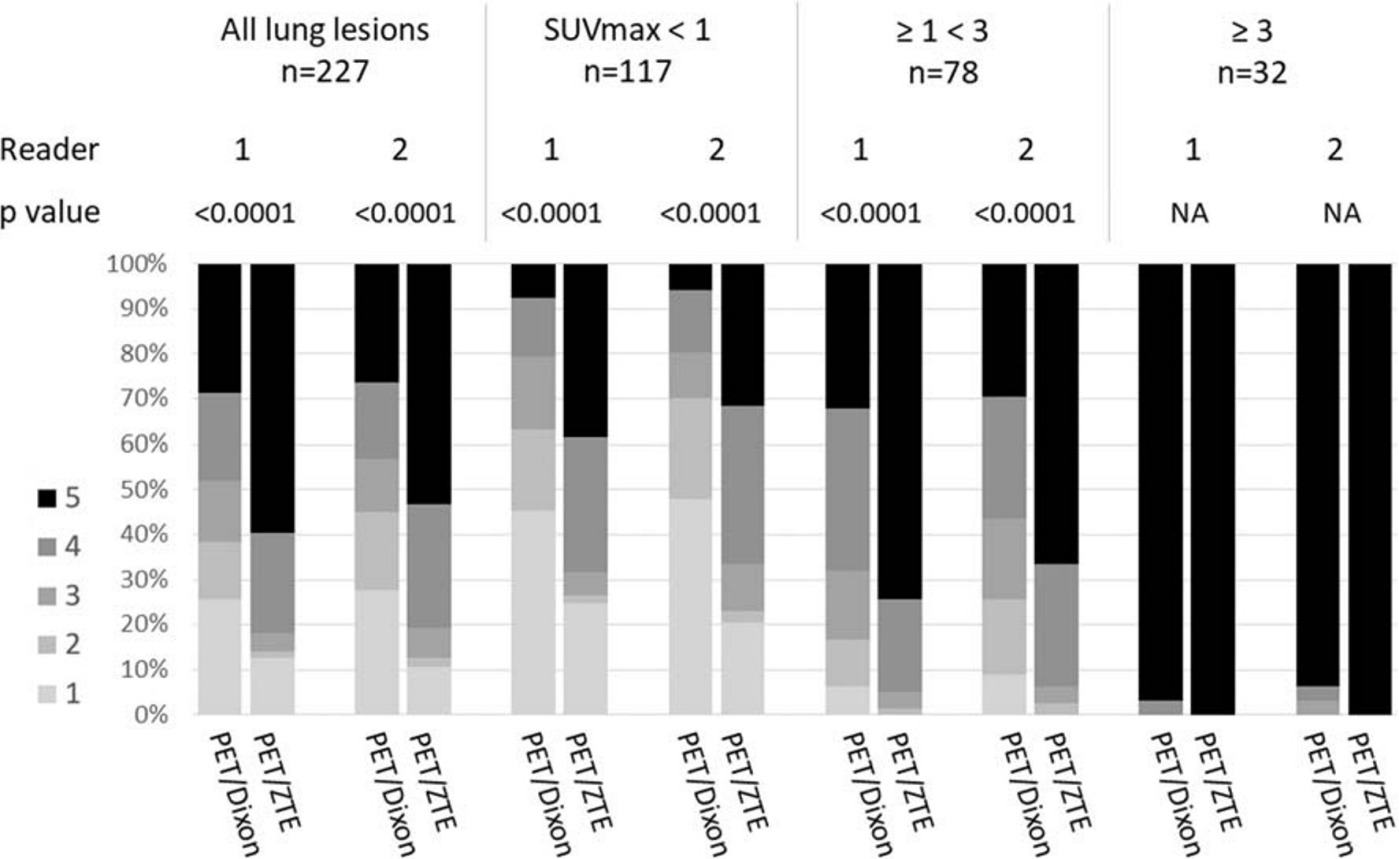
Stocked bar chart representing the distribution of percentages of the detectability scores using PET/Dixon and PET/ZTE in all lesions and in each subgroup based on lesion SUVmax for both readers. The detectability was significantly superior in PET/ZTE for lesions with SUVmax less than 3 (*p* < 0.0001)

The detectability scores obtained by PET/Dixon and PET/ZTE for the overall sample and each subgroup based on SUVmax are shown in Fig. 3. The detectability score with PET/ZTE was significantly higher than that with PET/Dixon for all lung lesions for both readers (*p* < 0.0001). In the subgroups based on lesion SUVmax, the detectability score of PET/ZTE was significantly higher than that of PET/Dixon for lesions with SUVmax less than 3 (*p* < 0.0001, Supplemental File).

**Fig. 4:**
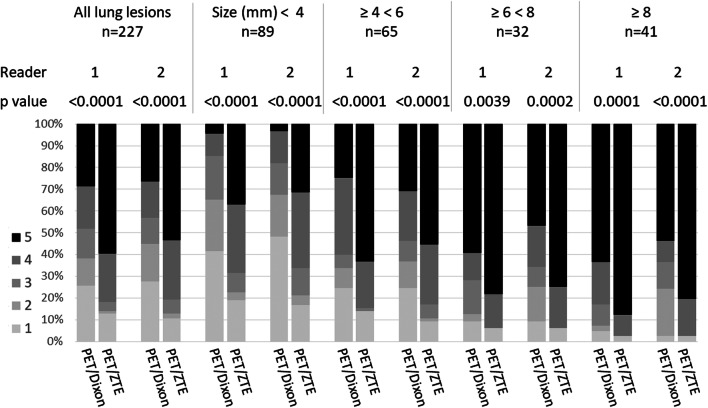
Stocked bar chart representing the distribution of percentage of the detectability scores by PET/Dixon and PET/ZTE in all lesions and each subgroup based on lesion size for both readers. The detectability by PET/ZTE was significantly superior for all subgroups (*p* < 0.004)

The detectability of the overall lesion sample and subgroup analysis based on size are shown in Fig. 4 and Supplemental File. The detectability score of PET/ZTE was significantly higher than that of PET/Dixon for all subgroups based on size in assessments by both readers (*p* < 0.004, Supplemental File).

### Lesion-based analysis: differentiation of lung lesions

**Fig. 5:**
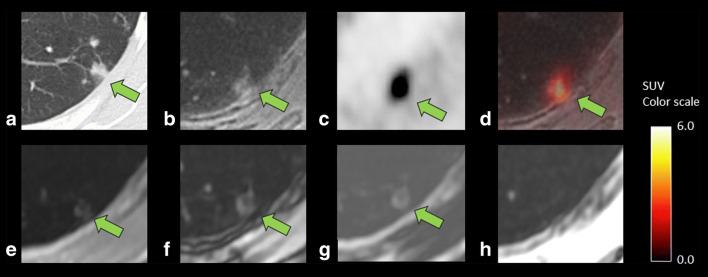
A case showing lung inflammatory changes in a 75-year-old woman after surgery of pancreatic cancer. CT (**a**) showed a 7.9-mm-sized lesion with a linear margin and a component of ground-glass opacity, which were concordant findings with an inflammatory process. ZTE (**b**) can clearly reveal these CT characteristics for the margin and the opacity, leading to correct diagnosis of active inflammatory lesions by combined findings of the high FDG uptake (SUVmax 6.9) on PET (**c**) and fused images (**d**). Dixon sequence (**e**, in phase; **f**, out of phase; **g**, fat; and **h**, water image, respectively), however, failed to depict those characteristics of the component of ground-glass opacity and simply showed a nodule-like lesion in comparison with ZTE, resulting in incorrect diagnosis of malignant nodule

**Fig. 6:**
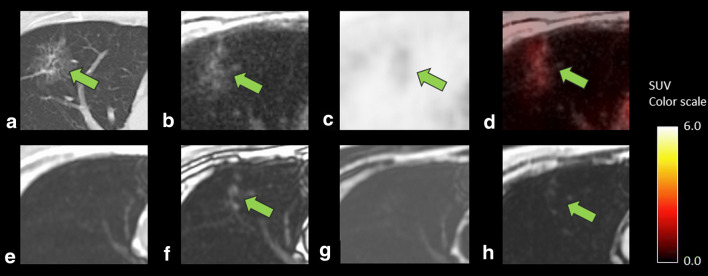
A case showing lung adenocarcinoma in a 61-year-old man after surgery for pharyngeal cancer. CT (**a**) showed a 7.3-mm-sized lesion with ground-glass opacity component. ZTE (**b**) can reveal these CT characteristics, leading to correct diagnosis of lung cancer by the combined findings of the FDG uptake on PET (SUVmax 3.9, **c**) and fused images (**d**). Dixon sequence (**e**, in phase; **f**, out of phase; **g**, fat; and **h**, water image, respectively), however, failed to depict the characteristics of ground-glass opacity, resulting in the diagnosis of differentiation as equivocal

The ROC curves are shown in Fig. 2, and the AUCs in each method in assessments by readers 1 and 2 are summarized in Table 2. For overall lesions, the AUC in PET/ZTE was significantly larger than that in PET/Dixon in assessments by both readers (reader 1, *p* = 0.0016; reader 2, *p* = 0.0001, Figs. 5 and 6). The AUC in PET/ZTE was significantly larger than that in PET/Dixon in assessments by reader 2 for the subgroups of FDG non-avid lesion (*p* = 0.0004). The AUC in PET/ZTE was significantly larger than that in PET/Dixon for lung lesions smaller than 4 mm in diameter in assessments by both readers (reader 1, *p* = 0.0012; reader 2, *p* = 0.0006). Sensitivity and specificity of each method in assessments by readers 1 and 2 are shown in Table 3. When the cutoff value of score 3 was applied, the sensitivity of PET/ZTE was significantly higher than that of PET/Dixon in assessments by both readers (reader 1, *p* = 0.0078; reader 2, *p* = 0.001).

**Table 2:** The area under the ROC curve for all lesions and in subgroups based on the lesion SUVmax and size

Group	Reader	PET/Dixon	PET/ZTE	*p* value
All lung lesions	1	0.829 (0.774–0.876)	0.924 (0.881–0.955)	0.0016*
	2	0.821 (0.765–0.869)	0.943 (0.904–0.969)	0.0001*
SUVmax < 1	1	0.530 (0.435–0.623)	0.784 (0.698–0.854)	0.0912
	2	0.561 (0.466–0.652)	0.892 (0.821–0.942)	0.0004 *
SUVmax 1–3	1	0.910 (0.823–0.963)	0.952 (0.878–0.988)	0.0132
	2	0.869 (0.773–0.935)	0.937 (0.859–0.980)	0.1033
SUVmax ≥ 3	1	0.866 (0.699–0.960)	0.881 (0.717–0.968)	0.1936
	2	0.771 (0.588–0.900)	0.887 (0.726–0.971)	0.0261
Size < 4 mm	1	0.586 (0.477–0.689)	0.879 (0.792–0.938)	0.0012 *
	2	0.628 (0.519–0.728)	0.950 (0.883–0.985)	0.0006 *
Size 4–6 mm	1	0.887 (0.784–0.952)	0.911 (0.814–0.967)	0.0738
	2	0.872 (0.766–0.942)	0.914 (0.818–0.969)	0.0234
Size 6–8 mm	1	0.915 (0.761–0.984)	0.947 (0.806–0.995)	0.1492
	2	0.891 (0.730–0.973)	0.998 (0.887–1.000)	0.0230
Size ≥ 8 mm	1	0.963 (0.852–0.997)	0.981 (0.880–1.000)	0.1586
	2	0.884 (0.745–0.963)	0.918 (0.789–0.980)	0.3160

*Statistically significant difference

Numbers in parentheses are 95% confidence intervals

**Table 3:** Sensitivity and specificity for the differentiation of all lung lesions by each method

Variable	Reader	PET/Dixon	PET/ZTE	*p* value
Sensitivity	1	59.3% (32/54)	74.1% (40/54)	0.0078 *
	2	63.0% (34/54)	83.3% (45/54)	0.0010 *
Specificity	1	98.8% (171/173)	98.8% (171/173)	> 0.05
	2	97.1% (168/173)	98.8% (171/173)	0.2500

*Statistically significant difference

The weighted Cohen’s kappa coefficients for inter-rater variability of differentiation for all lesions and each subgroup are summarized in Table 4. The weighted Cohen’s kappa coefficients for inter-rater variability of differentiation for all lesions and all subgroups in PET/ZTE were larger than those in PET/Dixon. For subgroup analysis with SUVmax, the inter-rater variability tended to improve as the SUVmax increased in PET/ZTE.

**Table 4:** Cohen’s kappa coefficients for inter-rater variability of differentiation score in all lesions and in subgroups, based on the lesion SUVmax and size

Group	PET/Dixon	PET/ZTE
All lung lesions	0.641 (0.569–0.713)	0.723 (0.663–0.783)
SUVmax < 1	0.577 (0.459–0.695)	0.591 (0.455–0.727)
SUVmax 1–3	0.516 (0.375–0.658)	0.635 (0.528–0.743)
SUVmax ≥ 3	0.582 (0.402–0.762)	0.792 (0.668–0.916)
Size < 4 mm	0.580 (0.427–0.734)	0.650 (0.523–0.778)
Size 4–6 mm	0.653 (0.533–0.773)	0.745 (0.646–0.844)
Size 6–8 mm	0.659 (0.495–0.823)	0.718 (0.589–0.846)
Size ≥ 8 mm	0.551 (0.378–0.724)	0.686 (0.536–0.837)

Numbers in parentheses are 95% confidence intervals

## Discussion

Our results showed that ZTE in combination with PET provided higher performance than the conventional Dixon sequence in lesion detection for non-FDG-avid lung lesions as well as in nodules smaller than 4 mm in diameter. In addition to lesion detectability, PET/ZTE showed superior diagnostic performance in differentiating lung lesions compared to PET/Dixon, which was also observed for the non-FDG-avid lesions by all readers and small-sized lesions by the experienced reader. The published data evaluated only the detectability but not the differentiation ability [8].

Several studies have evaluated the detection of lung lesions using PET/MRI. The detection rates of simultaneously acquired VIBE sequence and PET on PET/MRI for small lung lesions less than 1 cm in diameter were 45.4% [13]. The sensitivity of PET/MRI with the radial VIBE sequence was 88.6% for nodules 5 mm in diameter or larger. The detectability was higher on respiratory-gated T2-PROPELLER (60%) compared to T1-weighted Dixon-type sequences (16.1–37.8%). In our results, the detectability (score 4 or 5) with ZTE was more than 80% for lung lesions 4 mm or larger in diameter, and more than 93% for lesions 6 mm or larger in diameter in assessments by both readers, suggesting better performance than the previously reported MR sequences. The detectability of FDG-avid nodules was significantly higher on radial VIBE (95.6%) and respiratory-gated T2-PROPELLER (92.4%) [14, 15], which was concordant with our results.

Conventional MR techniques are not suitable for delineation of lung lesion characteristics in comparison with CT, which is a major drawback of using MRI to capture essential information for differentiating benign and malignant lesions. This drawback is mainly caused by the low proton density in the lung as well as respiratory and cardiac motion in the thorax. Cieszanowski et al reported that the sensitivity for detection of lung lesions in out-of-phase Dixon images was 48.7% [16]. De Galiza Barbosa F et al showed that the detectability of lung nodules in Dixon sequences was 29.4% in water, 16.1% in fat, 33.8% in in-phase images and 37.8% in out-of-phase images [15]. Because echo time (TE) of the out-of-phase images was the shortest among the four Dixon images, the performance for delineation of lung lesions was considered to be highly dependent on how short the TE was.

Using a very short TE like ultra-short echo time (UTE) and the ZTE sequence is effective in acquiring lung signal intensity [17]. A UTE sequence therefore shows high sensitivity for detection of small pulmonary nodules and is superior to the three-dimensional dual-echo GRE technique for detection of small, non-FDG-avid nodules. The detection rate of pulmonary nodules 4 mm or larger in diameter using UTE was 82% [9], similar to our results obtained using ZTE. Because of the surrounding lung tissue (i.e. air), delineation of the peripheral structures is hampered by the very short T2* relaxation time, particularly under high magnetic field strength [18, 19]. ZTE can provide more structural information of the lung in high resolution with a better signal-to-noise ratio and contrast-to-noise ratio than UTE (owing to the shorter echo time), resulting in superior image quality to UTE for lung parenchymal structures [8]. In our study, lung lesions with a ground-glass opacity component were appreciated by ZTE and could be diagnosed as a lung cancer on PET/ZTE. In contrast, Dixon could not reveal the characteristics of the lesion, resulting in diagnosis of differentiation as equivocal.

Our results revealed that the inter-rater variability with PET/ZTE was better than that with PET/Dixon even for differentiating nodules smaller than 4 mm in diameter and/or with low FDG uptake for experienced readers, suggesting that the concordance rate for the diagnosis of small nodules and/or non-FDG-avid lesions between novice and experienced readers may improve with PET/ZTE fusion imaging.

Our study had some limitations. First, CT images as reference standards were obtained with breath-holding in deep inspiration, whereas PET/MR images were acquired with respiratory gating under free-breathing performed on a different date from CT, resulting in possible discrepancies between reference-standard CT and MRI. Although breath-holding in deep inspiration is an ideal protocol for lung imaging, it is difficult to obtain both PET and MR images with breath-holding in deep inspiration and create precisely fused images. In this regard, registration of fused images acquired simultaneously in the same respiratory cycle (shallow expiratory phase) on PET/MRI may be more precise than that of images acquired separately in different respiratory cycles on PET/CT, but further investigation beyond our study is necessary to confirm the issue. Second, for clinical and ethical reasons, 223 reference standards for the lung lesions were not confirmed by pathological findings but by radiological follow-up examinations. Due to the relatively limited follow-up durations, accurate final diagnosis as a reference standard could not be obtained for the entire study population. Third, because direct comparison between PET/CT and PET/MRI was not possible in our study, evaluation of the advantages of PET/MRI over PET/CT was not performed and warrants further investigation. Fourth, reader 1 analyzed the reference standard as well as PET/MRI data, which could introduce bias in the evaluation of the diagnostic performance. In order to minimize this bias, reader 1 conducted the scoring for the diagnostic test at 2-month intervals from the analysis the reference standard.

In conclusion, PET/ZTE outperforms PET/Dixon in terms of diagnostic performance for detection and differentiation of FDG-avid and non-FDG-avid lung lesions and can be a promising tool to enhance the utility of FDG PET/MRI in oncology patients with lung lesions.

## Electronic supplementary material

ESM 1 (DOCX 1504 kb).

## Abbreviations

AUC: Area under the curve
FA: Flip angle
FDG: ^18^F-Fluorodeoxyglucose
FOV: Field of view
NEX: Number of excitations
ROC: Receiver operating characteristic
SUV: Standardized uptake value
TE: Echo time
TOF-OSEM: Time-of-flight ordered subset expectation maximization
TR: Repetition time
UTE: Ultra-short echo time
ZTE: Zero echo time
