# Supplementary material for: Diagnostic performance of zero-TE lung MR imaging in FDG PET/MRI for pulmonary malignancies
Source: Eur Radiol. 2020 Apr 16;30(9):4995–5003. doi: 10.1007/s00330-020-06848-z (PMC7431435; doi:10.1007/s00330-020-06848-z)
Supplement: Supplementary file 1 — (DOCX 1504 kb). [file 330_2020_6848_MOESM1_ESM.docx]

| Supplemental Table 1: Numbers of lung lesions overall and in the subgroups divided by SUVmax for each detectability score |
| --- |
| Group Modality Reader Score 1 Score 2 Score 3 Score 4 Score 5 |
| All lung lesions (n=227) PET/Dixon 1 58 (25.6%) 29 (12.8%) 31 (13.7%) 44 (19.4%) 65 (28.6%) |
| 2 63 (27.8%) 39 (17.2%) 27 (11.9%) 38 (16.7%) 60 (26.4%) |
| PET/ZTE 1 29 (12.8%) 3 (1.3%) 9 (4.0%) 51 (22.5%) 135 (59.5%) |
| 2 24 (10.6%) 5 (2.2%) 15 (6.6%) 62 (27.3%) 121 (53.3%) |
| SUVmax < 1 (n=117) PET/Dixon 1 53 (45.3%) 21 (17.9%) 19 (16.2%) 15 (12.8%) 9 (7.7%) |
| 2 56 (47.9%) 26 (22.2%) 12 (10.3%) 16 (13.7%) 7 (6.0%) |
| PET/ZTE 1 29 (24.8%) 2 (1.7%) 6 (5.1%) 35 (29.9%) 45 (38.5%) |
| 2 24 (20.5%) 3 (2.6%) 12 (10.3%) 41 (35.0%) 37 (31.6%) |
| SUVmax 1 - 3 (n=78) PET/Dixon 1 5 (6.4%) 8 (10.3%) 12 (15.4%) 28 (35.9%) 25 (32.1%) |
| 2 7 (9.0%) 13 (16.7%) 14 (17.9%) 21 (26.9%) 23 (29.5%) |
| PET/ZTE 1 0 (0%) 1 (1.3%) 3 (3.8%) 16 (20.5%) 58 (74.4%) |
| 2 0 (0%) 2 (2.6%) 3 (3.8%) 21 (26.9%) 52 (66.7%) |
| SUVmax ≥ 3 (n=32) PET/Dixon 1 0 (0%) 0 (0%) 0 (0%) 1 (3.1%) 31 (96.9%) |
| 2 0 (0%) 0 (0%) 1 (3.1%) 1 (3.1%) 30 (93.7%) |
| PET/ZTE 1 0 (0%) 0 (0%) 0 (0%) 0 (0%) 32 (100%) |
| 2 0 (0%) 0 (0%) 0 (0%) 0 (0%) 32 (100%) |

Numbers in parentheses are percentages.

| Supplemental Table 2: Numbers of lung lesions overall and in the subgroups divided by lesion size in each detectability score |
| --- |
| Group Modality Reader Score 1 Score 2 Score 3 Score 4 Score 5 |
| All lung lesions (n=227) PET/Dixon 1 58 (25.6%) 29 (12.8%) 31 (13.7%) 44 (19.4%) 65 (28.6%) |
| 2 63 (27.8%) 39 (17.2%) 27 (11.9%) 38 (16.7%) 60 (26.4%) |
| PET/ZTE 1 29 (12.8%) 3 (1.3%) 9 (4.0%) 51 (22.5%) 135 (59.5%) |
| 2 24 (10.6%) 5 (2.2%) 15 (6.6%) 62 (27.3%) 121 (53.3%) |
| Size < 4 (n=89) PET/Dixon 1 37 (41.6%) 21 (23.6%) 18 (20.2%) 9 (10.1%) 4 (4.5%) |
| 2 43 (48.3%) 17 (19.1%) 13 (14.6%) 13 (14.6%) 3 (3.4%) |
| PET/ZTE 1 17 (19.1%) 3 (3.4%) 8 (9.0%) 28 (31.5%) 33 (37.1%) |
| 2 15 (16.9%) 4 (4.5%) 11 (12.4%) 31 (34.8%) 28 (31.5%) |
| Size 4 - 6 (n=65) PET/Dixon 1 16 (24.6%) 6 (9.2%) 4 (6.2%) 23 (35.4%) 16 (24.6%) |
| 2 16 (24.6%) 8 (12.3%) 6 (9.2%) 15 (23.1%) 20 (30.8%) |
| PET/ZTE 1 9 (13.8%) 0 (0%) 1 (1.5%) 14 (21.5%) 41 (63.1%) |
| 2 6 (9.2%) 1 (1.5%) 4 (6.2%) 18 (27.7%) 36 (55.4%) |
| Size 6 - 8 (n=32) PET/Dixon 1 3 (9.4%) 1 (3.1%) 5 (15.6%) 4 (12.5%) 19 (59.4%) |
| 2 3 (9.4%) 5 (15.6%) 3 (9.4%) 6 (18.8%) 15 (46.9%) |
| PET/ZTE 1 2 (6.2%) 0 (0%) 0 (0%) 5 (15.6%) 25 (78.1%) |
| 2 2 (6.2%) 0 (0%) 0 (0%) 6 (18.8%) 24 (75.0%) |
| Size ≥ 8 (n=41) PET/Dixon 1 2 (4.9%) 1 (2.4%) 4 (9.8%) 8 (19.5%) 26 (63.4%) |
| 2 1 (2.4%) 9 (22.0%) 5 (12.2%) 4 (9.8%) 22 (53.7%) |
| PET/ZTE 1 1 (2.4%) 0 (0%) 0 (0%) 4 (9.8%) 36 (87.8%) |
| 2 1 (2.4%) 0 (0%) 0 (0%) 7 (17.1%) 33 (80.5%) |

Numbers in the parentheses are percentages.


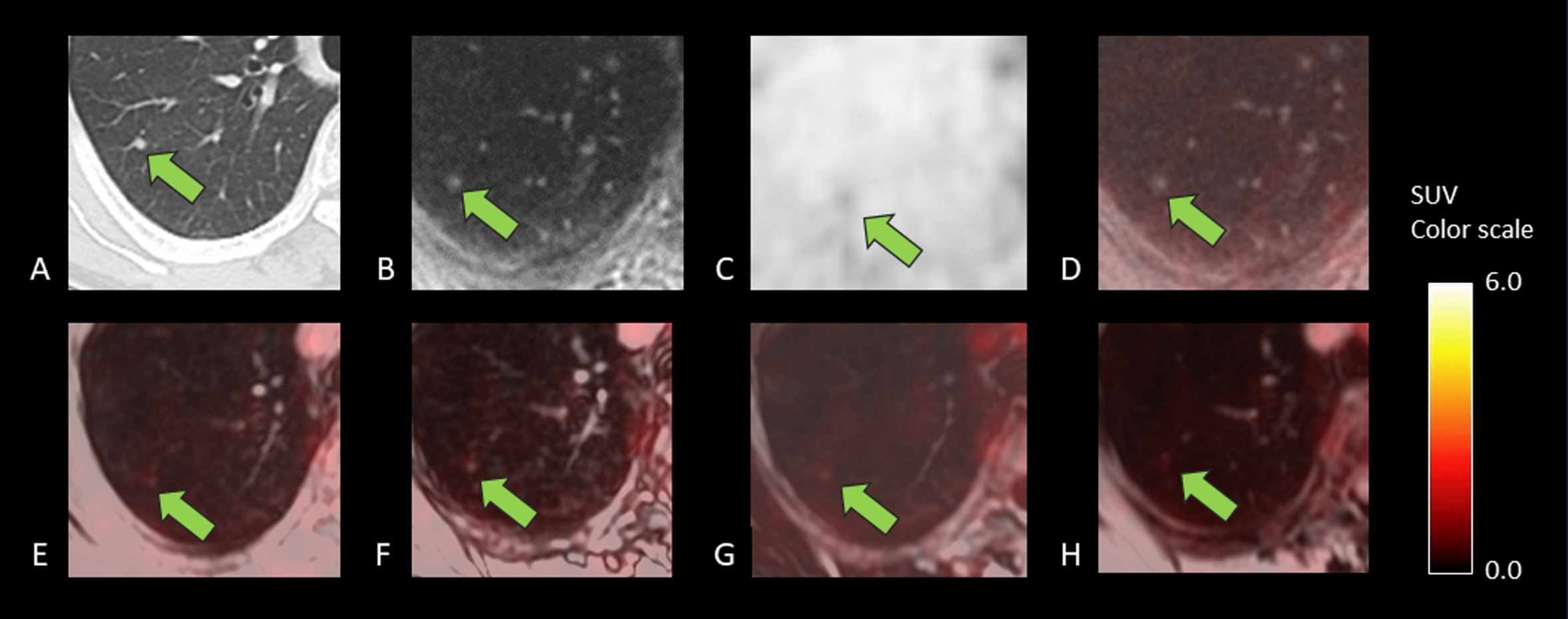


**Supplemental Figure:** A case showing a metastatic lung nodule of ovarian cancer in a 75-year-old woman with a diameter of 3.0 mm (arrows). The nodule was clearly detectable both on chest CT (A) and ZTE (B). PET (C) and PET/ZTE fused images (D) showed minimal FDG uptake with SUVmax of 0.92. On PET/Dixon fused images (E, in phase; F, out of phase; G, fat and H, water image, respectively), the corresponding nodule was ambiguous and mimicked a cross-section of bronchovascular bundle.
